# Assessing soil microbes that drive fairy ring patterns in temperate semiarid grasslands

**DOI:** 10.1186/s12862-022-02082-x

**Published:** 2022-11-05

**Authors:** Jiahuan Li, Lizhu Guo, Gail W. T. Wilson, Adam B. Cobb, Kun Wang, Li Liu, Huan Zhao, Ding Huang

**Affiliations:** 1 College of Grassland Science and Technology, West Campus of China Agricultural University, Haidian District, Beijing, 100193 China; 2 Horticultural College, Shenyang Agricultural University, Shenyang, 110866 China; 3 Institute of Grassland, Flowers and Ecology, Beijing Academy of Agriculture and Forestry Sciences, Beijing, 100097 China; 4 Oklahoma State University, 008C AGH, Stillwater, OK 74078 USA; 5 Academy of Inventory and Planning, National Forestry and Grassland Administration, Beijing, 100714 China

**Keywords:** Steppe grasslands, Biotic and abiotic factors, Plant–soil–microbial interactions, P-release, Microbial direct stimulation, Pathogen accumulation

## Abstract

**Background:**

Fairy rings occur in diverse global biomes; however, there is a critical knowledge gap regarding drivers of fairy rings in grassland ecosystems. Grassland fairy rings are characterized belowground by an expanding mycelial front and aboveground by vigorous vegetation rings that develop concentrically with each growing season. We evaluated fairy ring dynamics in a field study conducted in semiarid grasslands to elucidate above- and belowground interactions driving distinct vegetation patterns. We followed this initial field investigation with a complementary greenhouse experiment, using soils collected from specific fairy ring zones (inside, ring-edge, outside) to examine plant-soil-microbial interactions under controlled conditions. We selected *Leymus chinensis* (a dominant grass) as our model plant species to assess the role of diverse fairy ring microbial communities on plant growth and nutrition.

**Results:**

In our field study, plants on the ring-edge produced greater shoot biomass with higher concentrations of N and P, compared to plants inside the ring or adjacent (outside) controls. Soil microbial community biomarkers indicate shifts in relative microbial biomass as fairy rings expand. Inside the ring, plant roots showed greater damage from pathogenic fungi, compared to outside or ring-edge. Our greenhouse experiment confirmed that inoculation with live ring-edge soil generally promoted plant growth but decreased shoot P concentration. Inoculation with soil collected from inside the ring increased root pathogen infection and reduced shoot biomass.

**Conclusion:**

We propose that soil microbial activity within ring-edges promotes plant growth via mobilization of plant-available P or directed stimulation. However, as the ring expands, *L. chinensis* at the leading edge may increase pathogen accumulation, resulting in reduced growth at the center of the ring in subsequent growing seasons. Our results provide new insights into the plant-soil-microbial dynamics of fairy rings in grasslands, helping to elucidate these mysterious vegetation patterns.

**Supplementary Information:**

The online version contains supplementary material available at 10.1186/s12862-022-02082-x.

## Background

Fairy rings are enigmatic, frequently occur in grasslands dominated by clonal perennial gramineous plants, and are characterized by ring-like lush vegetation patterns [1, 2]. Grassland fairy rings, generally attributed to fungal activity, typically expand outward every growing season, with temperature and total precipitation influencing the rate of expansion [3]. Over 60 species of soil fungi, largely within *Basidiomycota* were detected in previous fairy ring research [1, 4]. The relationship between plant growth and soil microbial activity may drive fairy ring patterns, as fungal mycelium mineralize nutrients each season, facilitating plant growth on the ring-edge as the mycelial network expands [5]. Yang et al. suggested optimal soil N:P ratios, resulting from fairy ring fungal activity, promote plant growth on the ring-edge [2]. Furthermore, lush growth of vegetation along the ring-edge may be driven by expanding fungal mycelium, producing plant-growth promoting metabolites and hormones such as 2-azahypoxanthine (AHX) and Indole acetic acid (IAA) [1, 6, 7]. In addition, a consortium of other beneficial microbial functional groups may contribute to greater plant production along fairy ring-edges, however, few previous studies have focused on these soil microbes driving grassland fairy rings. For example, arbuscular mycorrhizal (AM) fungi are known to enhance host-plant nutrient access and stress tolerance, nearly 78% of vascular plant species associate with AM fungi, particularly in grasslands [8], as AM fungi increase plant growth in low-nutrient soils [9]. This widespread and beneficial symbiont is rarely included in fairy ring research, which primarily focuses on *Basidiomycota*. Zotti et al. assessed AM fungi using next generation sequencing and suggested a positive relationship between increased AM fungi and lush vegetative growth in fairy ring edges [10]. However, Zotti et al. did not find indication of AM fungal root colonization, which is the key indicator of plant–AM fungal symbioses.

Corresponding to the lush plant ring belt, poorly performing plants near the center or outside areas are common features of fairy rings. Direct pathogenic damage or cyanide released by fungi may the main factors harming plants in the inner area of fairy rings, resulting in reduced plant growth at fairy-ring centers [11]. Models of biomass dynamics indicate negative plant-soil feedbacks, driven by accumulated litter toxicity, suppress plant growth inside expanding rings [12, 13]. As a powerful driver of negative plant-soil feedbacks, soil-borne pathogens frequently decrease plant production in numerous ecosystems. Lush plant growth at the ring edge may accumulate pathogens, with a concomitant reduction in growth in subsequent years in the ring center. However, few empirical studies have assessed the influence of pathogenic organisms on fairy ring dynamics in temperate semiarid grasslands.

Numerous biotic or microbial-induced abiotic changes likely drive fairy ring dynamics, such as soil physical, chemical, and/or biological properties; however, these complex interactions have not been disentangled in controlled studies. Plant–microbial interactions (PMI) have received substantial attention in recent decades [14], and soil inoculation methods used in PMI experiments can help elucidate soil biotic and abiotic factors contributing to fairy ring dynamics. We utilized these controlled experimental methods to gain insight into grassland fairy ring patterns observed in the field.

We conducted two experiments to investigate (1) the distinct roles of soil microbial composition as well as potential changes to soil nutrient availability linked to fairy ring ecological patterns; (2) whether mycorrhizal fungi and/or pathogens have an influence on vegetation patterns associated with fairy rings.

## Methods

### Field investigation and sampling

**Fig. 1:**
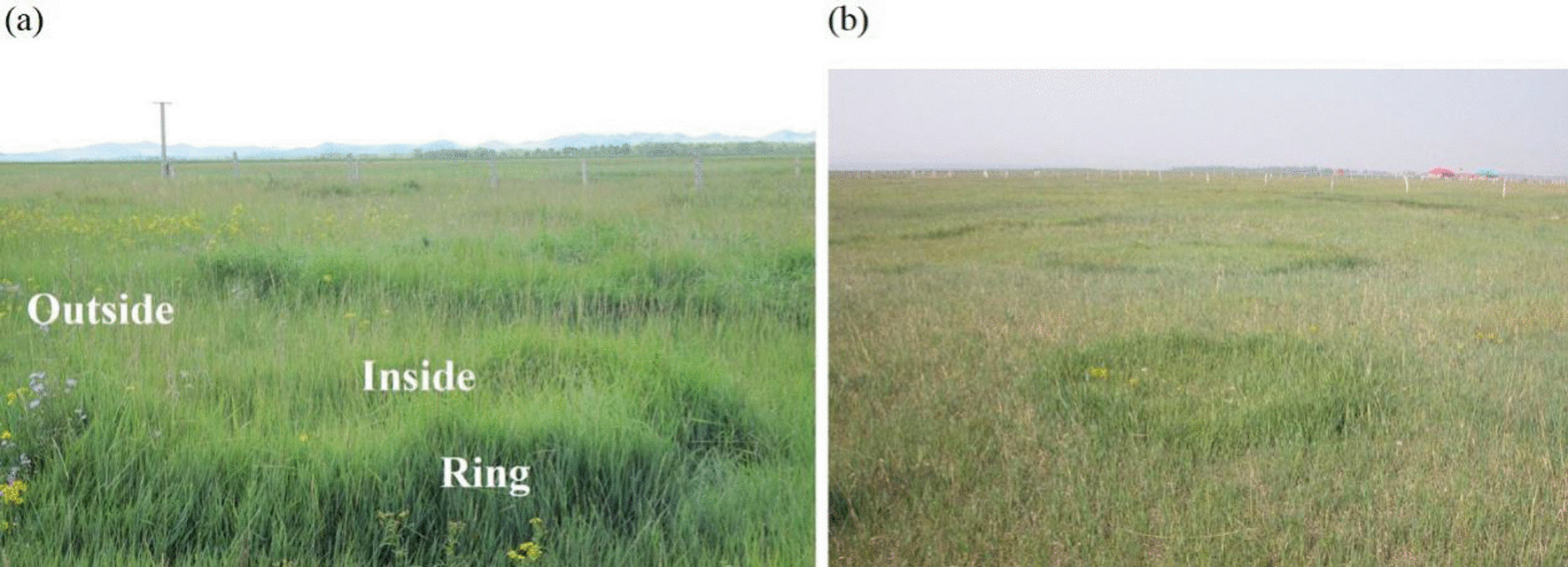
Photos of fairy rings taken at peak growing season in August. **a** Three fairy ring sampling areas used for field investigation and controlled microbial experiment (inside, ring-edge, and outside [control]) (Summer). **b** Overview of our study site in northern China, demonstrating fairy ring ecological patterns (Autumn)

Our field study was conducted at the National Field Station of Grassland Ecosystem (Guyuan, Hebei province, China, 41°46′ N, 115°40′E). Grazing is the primary utilization of these grasslands. Prior to our study, *Leymus chinensis* reseeding was carried out in 2000, with *L. chinensis* consistently accounting for > 40% of total aboveground biomass. Fairy rings in these grasslands are characterized by lush circular vegetation patches (Fig. 1). We selected three fairy rings in August 2017. Within each fairy ring, the diameter of the inside area was 3.5–5 m across, and the ring edge was defined by a 50–80 cm wide strip. Fairy rings were divided into three sampling areas: inside, ring-edge, and outside [adjacent control] (see Fig. 1). In each area, 8 soil samples were collected to a depth of 20 cm with a 20 cm diameter cylindrical core sampler, soil from each area in the same fairy ring was homogenized and analyzed as a single sample or used as inoculum in our greenhouse mesocosm study. Three fairy ring were treated as three blocks. Soils for microbiological analyses (20 mL in a certain area of each fairy ring) and future use in greenhouse (inoculation) (5 L in a certain area of each fairy ring) were stored at − 20 ℃. Soils for use as greenhouse substrate were dried at room temperature (20 L in a certain area of each fairy ring). Soils for chemical analyses were dried at 65 ℃ (1 L in a certain area of each fairy ring). Biomass of microbial functional groups (gram-positive and gram-negative bacteria; saprophytic and AM fungi), and total microbial biomass were assessed using phospholipid fatty acid analyses (PLFA), indicating relative abundances of each microbial functional group. As constituents of biological membranes, PLFA is widely applied to estimate active biomass of fungi and bacteria, as biovolume and cell surface area are well correlated [15]. Soil samples in close proximity to the plant roots were collected and homogenized. Fatty acids were extracted using a modification of the Bligh and Dyer extraction [16]. Qualitative and quantitative PLFA analyses were performed using gas chromatography with a GCMS unit Agilent 6890 gas chromatograph (Agilent Technologies, Palo Alto, CA, USA) and Sherlock software (MIDI Inc., Newark, NJ, USA). Biomarker c:19 was utilized as an internal standard. Biomarkers used to represent specific microbial functional groups were as follows: i14:0, 14:0, a15:0, i15:0, 15:0, i16:0, 16:1ω7, 16:0, 2-OH 16:0, a17:0, i17:0, i17:1, cy17:0, 18:1ω7c 18:0, and cy19:0 correspond to bacterial biomass; Saprotrophic fungal markers included 18:2ω2c and 18:1 ω9c; 16:1 ω5c was selected for AM fungal biomass [17, 18]. The composition of soil microbial communities was summarized using a correspondence analysis on the relative mole abundances of PLFAs in each sample [19]. Concentration of each individual functional group was calculated by summing corresponding selected biomarkers of each group.

Shoot density and biomass of *L. chinensis* were measured in three 0.04 m^2^ quadrates (0.2 m × 0.2 m) in each sampling area. Plants shoots were harvested and dried for 48 h at 65 °C. Plant samples were weighed and analysed for total aboveground tissue N and P. Fifty 1-cm root segments of *L. chinensis* were collected to estimate root disease and AM fungal root colonization. Many potentially pathogenic organisms may damage plant roots. To provide an estimate of total microbial damage, regardless of pathogen identity, we utilized methods of Schnitzer et al. [20], visually quantifying the incidence of root disease. Colonization by AM fungi was estimated using Trypan blue staining [21].

### Greenhouse experiment

Our complementary greenhouse experiment was designed to disentangle distinct influences of soil nutrients and plant–soil–microbial interactions in fairy ring vegetation patterns, building on field observations. We utilized a randomized block design, consisting of fully factorial combinations of three fairy ring zone substrates (collected from inside, ring-edge, and outside fairy rings) that were sterilized and three live fairy ring zone substrates to serve as inoculants (collected from inside, ring-edge, and outside rings) to elucidate the influences of biotic (microorganisms) and abiotic (nutrient availability) factors on fairy ring vegetation patterns. For each block, we established microcosms (12 cm deep, 6.5 cm bottom diameter; 10 cm top diameter) with a mixture of sterilized substrate soil, sterilized fine vermiculite, and a live soil inoculum treatment (4:2:1 V: V: V, total 500 mL). Each fairy ring was studied as an independent unit. For example, sterile fairy ring zone substrates from fairy ring A only received inoculant from fairy ring A. Fine vermiculite was added to ensure good drainage. With 3 blocks × 3 soil substrates × 3 inoculation treatments × 5 replicates, our experiment totalled 135 pots. Substrate soil was sieved (2 mm) and sterilized (Autoclaving, 121 °C, 103 kPa, 120 min). Soil inoculum was sieved (2 mm) and stored at − 20 °C prior to use.

Seeds of *L. chinensis* were field collected in autumn of 2016. Seeds were surface sterilized by washing for 5 min with 75% ethanol and then 5 min with 10% “84 disinfector” (dominant sector: NaClO, available chlorine 5.5–7%). Seeds were then rinsed with demineralized water, sown in 1-cm of wet sterilized soil, and placed in lighted growth chambers (16/8 h light/dark photoperiod, 26/20°C) to promote germination. After 15 days, two seedlings were transplanted into each pot. All pots were placed in the greenhouse (16/8 h light/dark photoperiod) at approximately 20–26 °C. Each pot was watered with 100 mL demineralized water and randomly rearranged every 5 days to ensure uniform conditions. Dead or unhealthy seedlings were replaced in the first week.

### Soil and plant analysis

Plant material and soil were analyzed for total nitrogen (N) content through combustion (FLASH.2000) and phosphorus (P) content through Mo-Sb-Vc spectrophotometry. In addition, soil was measured for extractable ammonium (${\text{NO}}_{3}^{-}$) and nitrate (${\text{NH}}_{4}^{+}$) by flow autoanalyzer (TRAACS-2000) and Olson P by Na_2_CO_3_ extraction. Soil saccharase and urease were measured by culturing and soil alkaline phosphatase was measured using Solarbio, BC0280 kit. Abundances of soil microbial biomarkers were assessed via phospholipid fatty acid analyses (described above).

Plants were harvested after 3 months and subsampled for microscopic assessments. Tissue was oven-dried at 65 °C for 48 h. We weighed shoot and root biomass and measured N and P tissue content. Root disease and AM fungal root colonization were measured, using the same method as used in our field experiment.

### Data analysis

For our field study, differences between plant and soil physicochemical properties and PLFA biomarkers between fairy ring sampling zones were analyzed using one-way ANOVA via SPSS 18.0, with ring zone as factors. Variation in soil microbial biomarkers was visualized by non-metric multidimensional scaling (NMDS) via “Vegan” in R [22].

For our greenhouse experiment, effects of fairy ring zone substrates and live fairy ring zone inoculations on plant performance were analyzed by two-way ANOVA, with live soil and soil substrate as fixed factors and block as a random factor.

## Results

### Field investigation

#### Plant characteristics

**Fig. 2:**
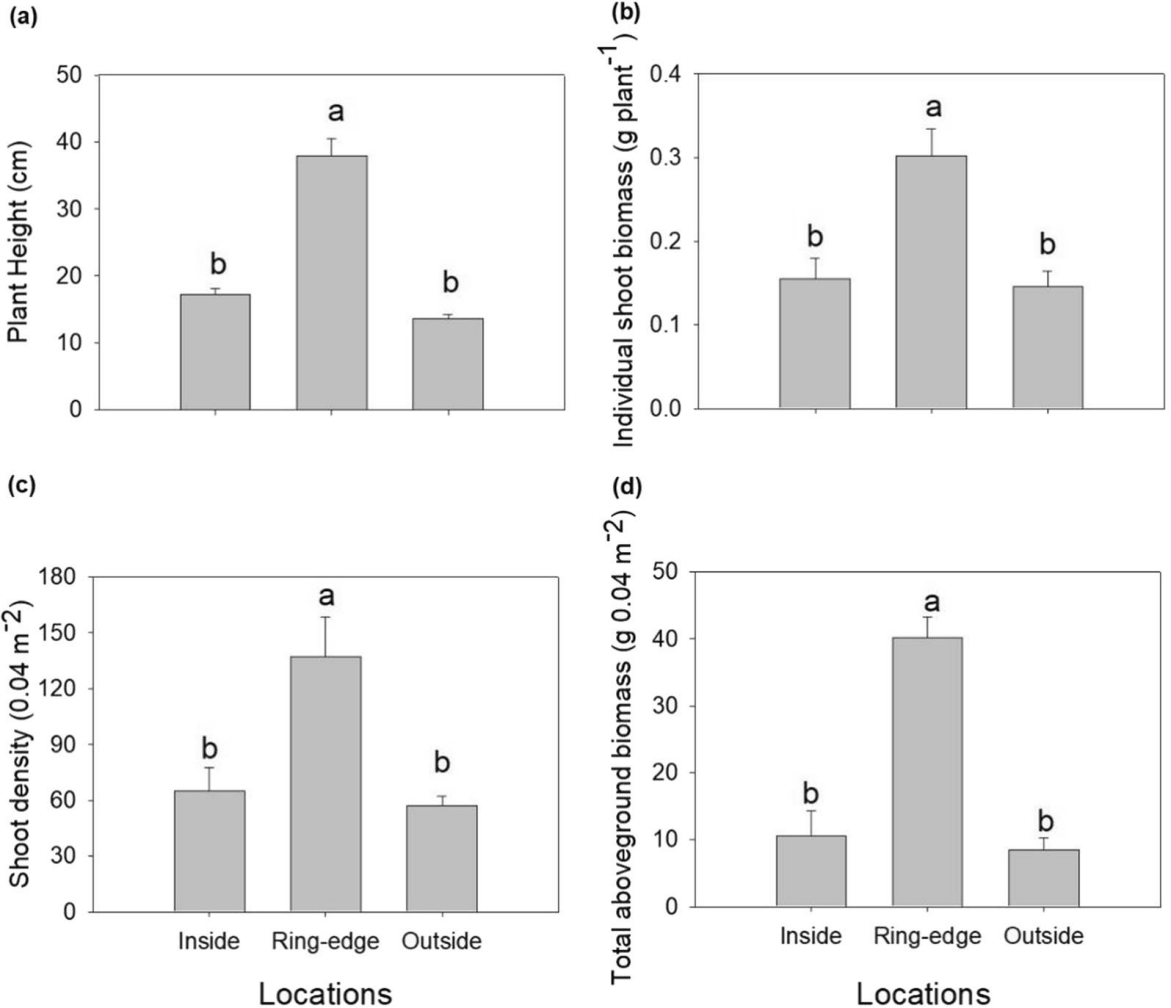
Plant height (**a**), individual shoot biomass (**b**), shoot density (**c**) and aboveground biomass in 0.04 m^2^ (**d**) at different fairy ring zones. Different letters indicate significant differences among three sampling zones (inside, ring-edge, and outside) (*p* < 0.05). Bars represent means + SE

**Fig. 3:**
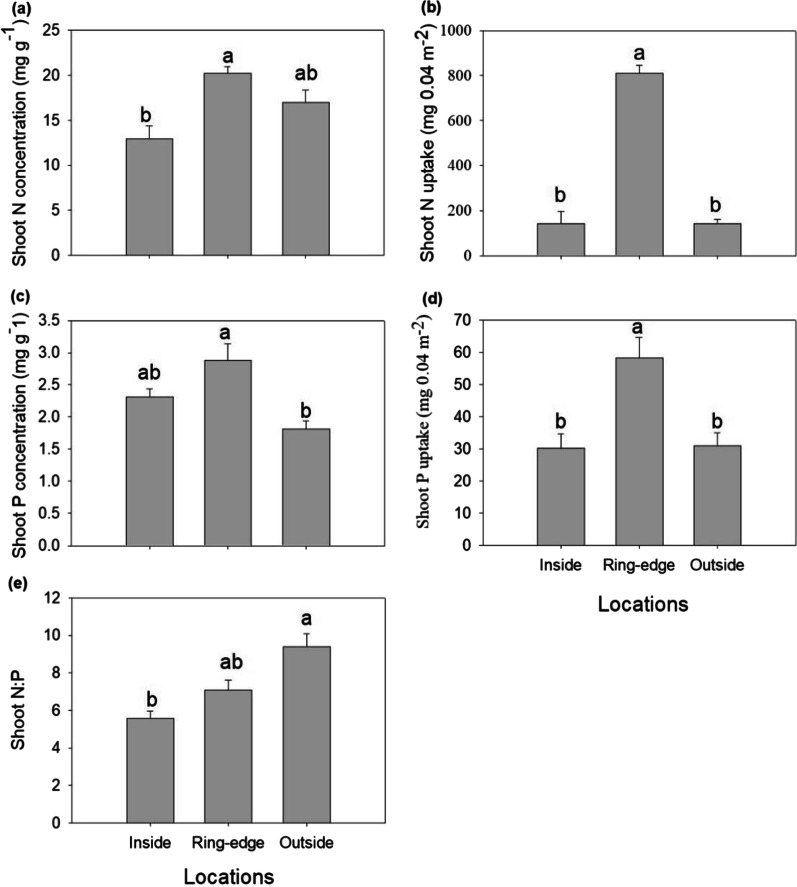
Shoot N concentration (**a**) and uptake (**b**), shoot P concentration (**c**) and uptake (**d**), and N:P ration (**e**) of plants harvested from three sampling zones (inside, ring-edge, and outside). Different letters indicate significant differences between three sampling areas (*p* < 0.05). Bars represent means + SE

Height, individual shoot biomass, shoot density, and total aboveground biomass of *L. chinensis* occurring inside and outside of each ring were each significantly less compared to plants on ring-edges (Fig. 2a–d). Shoot N and P concentrations of *L. chinensis* were highly variable among zones. Plants were characterized by lower shoot N concentration when growing at inside zones (Fig. 3a) and lowest in shoot P concentration when growing at outside zones (Fig. 3c). Plant N and P uptake were reduced on inside and outside zones, compared to plants growing on ring edges (Fig. 3bd). Shoot N:P ratio ranged from 5.56 to 9.39, with plants growing outside the rings lower than plants on inside zones (Fig. 3e).

#### Soil chemical properties and enzyme activities

Chemical properties and enzyme activities of soil at three fairy ring zones are presented in Table 1. Soil pH decreased from outside (8.98) to inside zones (8.25). Electric conductivity of soil from inside rings was lower than ring-edge and soil from outside rings. Soil total N (TN) and total P (TP) were not significantly different by zone. TN: TP ratio of outside soil was higher than inside or ring-edge soil. Ring-edge soil had highest plant-available nitrogen (AN). Plant-available P (AP) and alkaline phosphatase activity (APA) followed similar patterns: ring-edge soil ≥ inside soil > outside soil (AP: *p* = 0.011; APA: *p* = 0.003 ). Soil AN: AP ratio ranged from 4.96 to 7.49 with the lowest ratio occurring inside the fairy ring, increasing at ring-edge, and slightly decreasing outside the ring.

**Table 1:** Chemical properties and enzyme activities of soils at three sampling zones (inside, ring-edge, or outside)

	Inside	Ring-edge	Outside (control)
pH	8.25 ± 0.10c	8.58 ± 0.06b	8.98 ± 0.10a
EC (electric conductivity)	229.74 ± 35.39b	354.28 ± 24.13a	266.36 ± 10.46ab
Soil water content (%)	9.65 ± 0.73	8.94 ± 0.24	8.61 ± 0.03
Total N (µg g^− 1^ dry soil, TN)	2256.04 ± 260.27	2155.94 ± 206.02	2551.06 ± 347.90
Total P (µg g^− 1^ dry soil, TP)	481.02 ± 20.62	482.67 ± 21.16	490.89 ± 27.06
TN:TP ratio	4.66 ± 0.56	4.48 ± 0.49	5.20 ± 0.71
${\text{NH}}_{4}^{+}$-N (µg g^− 1^ dry soil)	11.77 ± 1.16b	29.46 ± 4.78a	9.14 ± 1.97b
${\text{NO}}_{3}^{-}$-N (µg g^− 1^ dry soil)	12.87 ± 3.37	7.57 ± 1.34	7.02 ± 3.79
AN (${\text{NH}}_{4}^{+}$-N + ${\text{NO}}_{3}^{-}$-N, µg g^− 1^ dry soil)	24.64 ± 2.45ab	38.37 ± 4.62a	16.17 ± 3.63b
Plant-available P (µg g^− 1^ dry soil, AP)	4.66 ± 0.47a	4.98 ± 0.33a	2.94 ± 0.14b
AN:AP ratio	4.96 ± 0.40	7.49 ± 0.90	5.53 ± 1.23
Saccharase activity (mg glucose g^− 1^ 24 h^− 1^)	38.59 ± 4.22	37.14 ± 5.75	34.39 ± 8.49
Urease activity (µg ${\text{NH}}_{3}^{-}$N g^− 1^ 3 h^− 1^)	12.19 ± 2.17	10.53 ± 1.51	11.68 ± 2.78
Alkaline phosphatase activity (µmol phenol g^− 1^ 24 h^− 1^)	22.50 ± 0.39ab	24.18 ± 0.34a	20.98 ± 0.424b

Different letters indicate significant difference in Duncan’s multiple range tests reported from one-way (*p* < 0.05)

#### Soil microbial community biomarkers

**Fig. 4:**
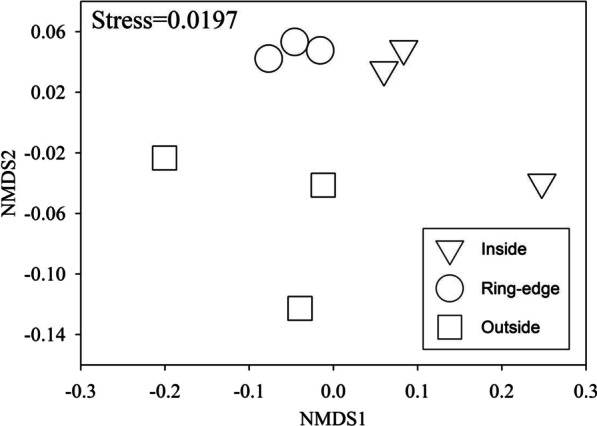
Soil microbial composition as indicated by nonmetric multidimensional scaling (NMDS) based on Bray-Curtis dissimilarity matrices across three sampling zones (inside, ring-edge, and outside), based on phospholipid fatty acid (PLFA) biomarkers (nmol g^− 1^ dry soil) extracted from soil. Sampling zones are represented by different shapes

Based on NMDS, soil microbial community biomarkers were highly variable (Fig. 4) across all three fairy zones, but generally homogeneous among each of the three fairy rings we assessed. Soil microbial community biomarkers in ring-edge soil separated from outside (control) soil. As fairy rings expand each season, soil microbial community biomarkers inside the rings show separation from microbial biomarkers of soils outside the rings. Results of PLFA analyses indicate significant differences by fairy ring zone (Table 2). Total PLFA biomarkers were 15.3% and 13.6% less abundant at inside zones compared to ring-edge and outside zones, respectively. Percentage of bacteria and AM fungal PLFA biomarkers of inside soil were significantly greater, compared to outside the rings. Saprotrophic fungal PLFA biomarkers of soil outside rings were 30.6% and 27.9% more abundant, compared to inside and ring-edge soil. Fungi: bacteria ratio showed a trend of decreasing from outside to inside zones (*p* = 0.078).

**Table 2:** Abundance of microbial functional groups (indicated by PLFA biomarkers); total microbial biomass, bacteria, saprophytic fungi, arbuscular mycorrhizal (AM) fungi, actinomycetes and fungal: bacterial ratio of three sampling zones (inside, ring-edge, and outside)

	Total (nmol g^− 1^ dry soil)	Bacteria (%)	Saprophytic (%)	AM fungi (%)	Actinomycetes (%)	F/B
Inside	32.35 ± 2.71	33.44 ± 0.55a	5.16 ± 0.19	2.11 ± 0.19a	2.62 ± 0.19	0.15 ± 0.0005
Ring-edge	38.19 ± 0.72	30.77 ± 0.63ab	5.27 ± 0.11	1.88 ± 0.03ab	2.72 ± 0.15	0.17 ± 0.005
Outside	37.45 ± 1.00	29.46 ± 0.55b	6.74 ± 0.85	1.53 ± 0.04b	2.74 ± 0.04	0.23 ± 0.03

Different letters indicate significant difference in Duncan’s multiple range tests reported from one-way (*p* < 0.05)

#### AM fungal colonization and root disease

**Fig. 5:**
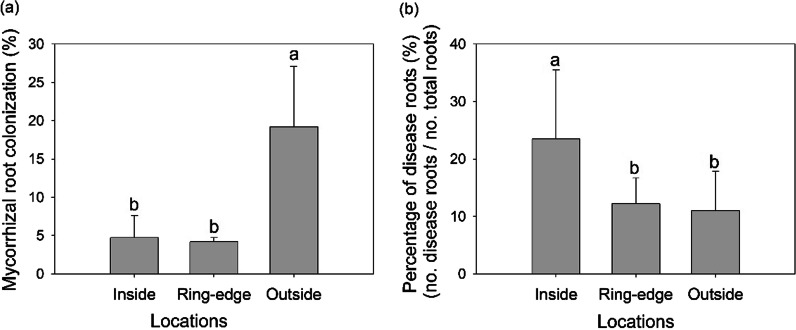
Arbuscular mycorrhizal (AM) fungal and pathogen root colonization at three sampling zones (inside, ring-edge, and outside) in the field. Root colonization by AM fungi (**a**) and pathogen colonization of diseased roots (no. diseased roots/ no. total roots) (**b**) are expressed in percentages. Bars represent means + SE

*L. chinensis* growing on the outside zone had 307.82% and 358.20% greater AM fungal root colonization than those grown on inside and ring-edges (*p* < 0.05) (Fig. 5a). The percentage of diseased roots showed the opposite trend, with roots at the inside zone having 92.0% and 113.0% more infection compared to ring-edge or outside zones (*p* < 0.05) (Fig. 5b).

### Greenhouse experiment

#### Nutrient availability of soil substrates

Plant-available N increased after substrate soil sterilization and ring-edge soil had the highest N concentration. Plant-available P of outside soil was significantly lower than ring-edge and inside soils. Plant-available N: P ratio decreased from outside to inside zones (Table 3).

**Table 3:** Plant-available N and P of soil collected from three sampling zones (inside, ring-edge, or outside) after sterilization (used in greenhouse experiment)

	Inside	Ring-edge	Outside
${\text{NH}}_{4}^{+}$-N (µg g^− 1^ dry soil)	29.15 ± 3.56	39.97 ± 5.44	28.56 ± 1.99
${\text{NO}}_{3}^{-}$-N (µg g^− 1^ dry soil)	127.82 ± 18.29	135.33 ± 9.61	113.44 ± 4.82
Plant-available N (${\text{NH}}_{4}^{+}$-N + and ${\text{NO}}_{3}^{-}$-N, µg g^− 1^ dry soil)	156.97 ± 14.86ab	175.31 ± 14.66a	141.99 ± 3.59b
Plant-available P (µg g^− 1^ dry soil, AP)	4.66 ± 0.30a	4.66 ± 0.23a	3.45 ± 0.14b
AN:AP ratio	34.02 ± 4.34	37.93 ± 4.63	41.14 ± 0.64

Different letters indicate significant difference in Duncan’s multiple range tests reported from one-way ANOVA (*p* < 0.05)

#### Plant biomass

**Fig. 6:**
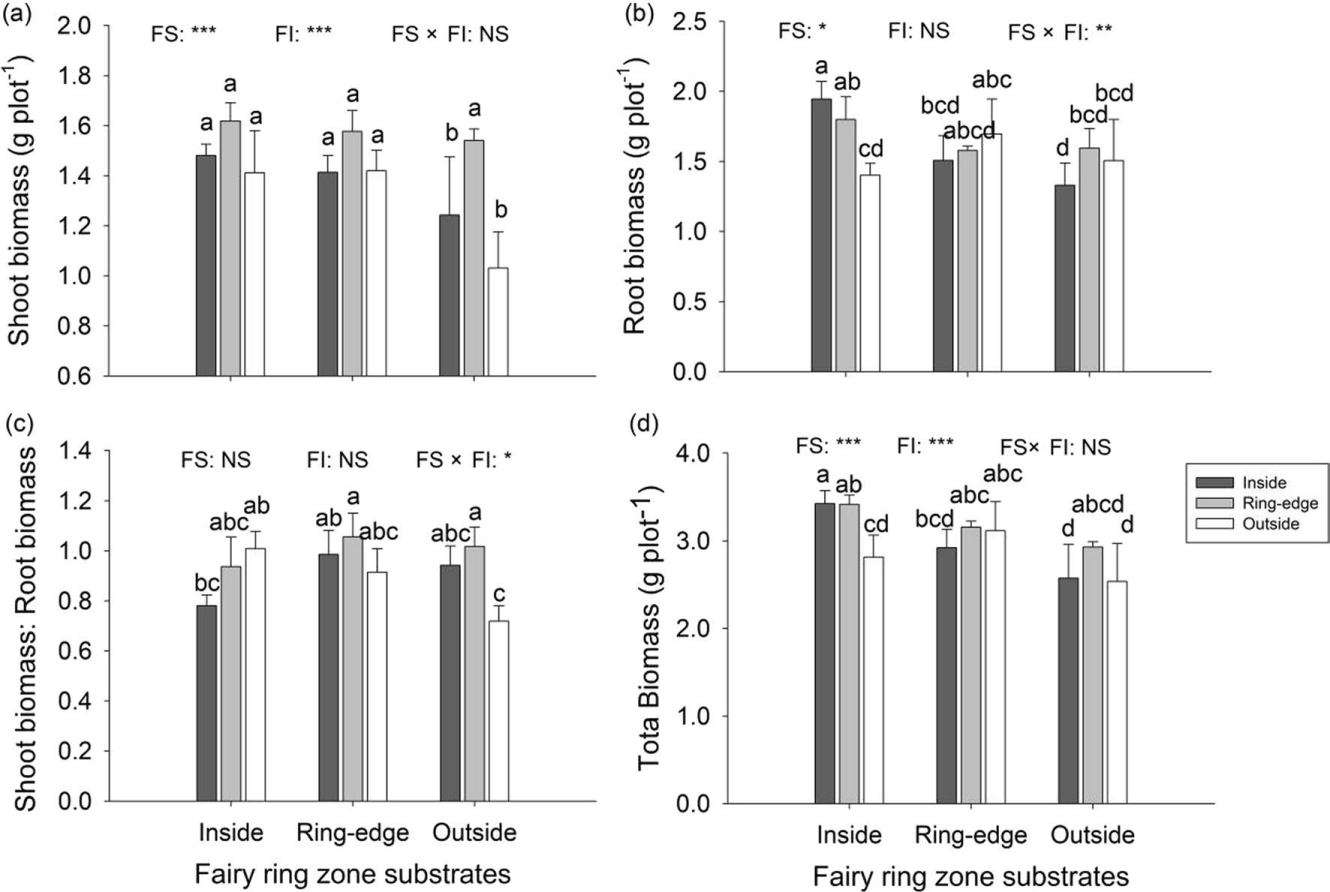
Biomass of *L. chinensis* grown in fairy ring zone substrates (collected from inside, ring-edge, and outside zones) inoculated with inside, ring-edge, or outside live soil (microorganisms) in greenhouse experiment. Bars represent means + SE. Different letters indicate significant differences between treatments (*p* < 0.05), reported by Duncan’s multiple range test from one-way ANOVA

Fairy ring zone substrates and live fairy ring zone inoculations each had significant effects on shoot biomass and total biomass, while their interactions were not significant (Table 4). Shoot biomass and total biomass of plants grown in outside substrate were less than plants grown in inside and ring-edge substrates, when pots were inoculated with live soil from either inside or outside the rings (Fig. 6a–d). Ring-edge soil inoculation increased plant shoot biomass and total biomass, especially on substrate from outside fairy rings (Fig. 6a–d). Plant root biomass was significantly different across fairy ring zone substrates, and also showed a significant interaction with fairy ring zone inoculations (Table 4). Shoot biomass: root biomass ratio showed significant substrates × inoculations interactions.

**Table 4:** Three-way analysis of variance (ANOVA) for the effects of fairy ring zone substrates, fairy ring zone inoculations, and interactions on plant growth and nutrient characteristics in greenhouse experiment, using blocks as random factors

		Fairy ring zone substrates (FS)			Fairy ring zone inoculations (FI)			FS × FI			Block		
		df	*F*	*p*	df	*F*	*p*	df	*F*	*p*	df	*F*	*p*
Shoot biomass		2	10.48	**< 0.001**	2	14.85	**< 0.001**	4	2.18	0.76	2	13.21	**< 0.001**
Root biomass		2	4.15	**0.018**	2	1.48	0.232	4	3.76	**0.007**	2	1.15	0.320
Shoot biomass: root biomass		2	1.62	0.205	2	2.13	0.124	4	3.50	**0.010**	2	2.07	0.131
Total biomass		2	9.49	**< 0.001**	2	4.08	**0.009**	4	1.94	0.108	2	4.90	**0.009**
Shoot N concentration (mg g^− 1^ dry soil)		2	6.10	**0.003**	2	13.76	**< 0.001**	4	1.52	0.204	2	6.71	**0.002**
Shoot N uptake (mg)		2	18.47	**< 0.001**	2	16.57	**< 0.001**	4	2.19	0.077	2	20.78	**< 0.001**
Shoot P concentration (mg g^− 1^ dry soil)		2	1.39	0.253	2	5.63	**0.005**	4	2.05	0.094	2	28.59	**< 0.001**
Shoot P uptake (mg)		2	16.29	**< 0.001**	2	5.97	**0.004**	4	1.33	0.265	2	8.01	**0.001**
Percentage of diseased roots (%)		2	0.154	0.857	2	1.82	0.167	4	0.59	0.674	2	3.95	**0.022**
Mycorrhizal root colonization (%)		2	0.064	0.938	2	0.54	0.582	4	0.53	0.715	2	0.075	0.928

Percentage of diseased roots was determined by number of diseased roots/number of total roots

Statistically significant sources of variation are in bold (*p* < 0.05)

#### Plant nutrient characteristics

**Fig. 7:**
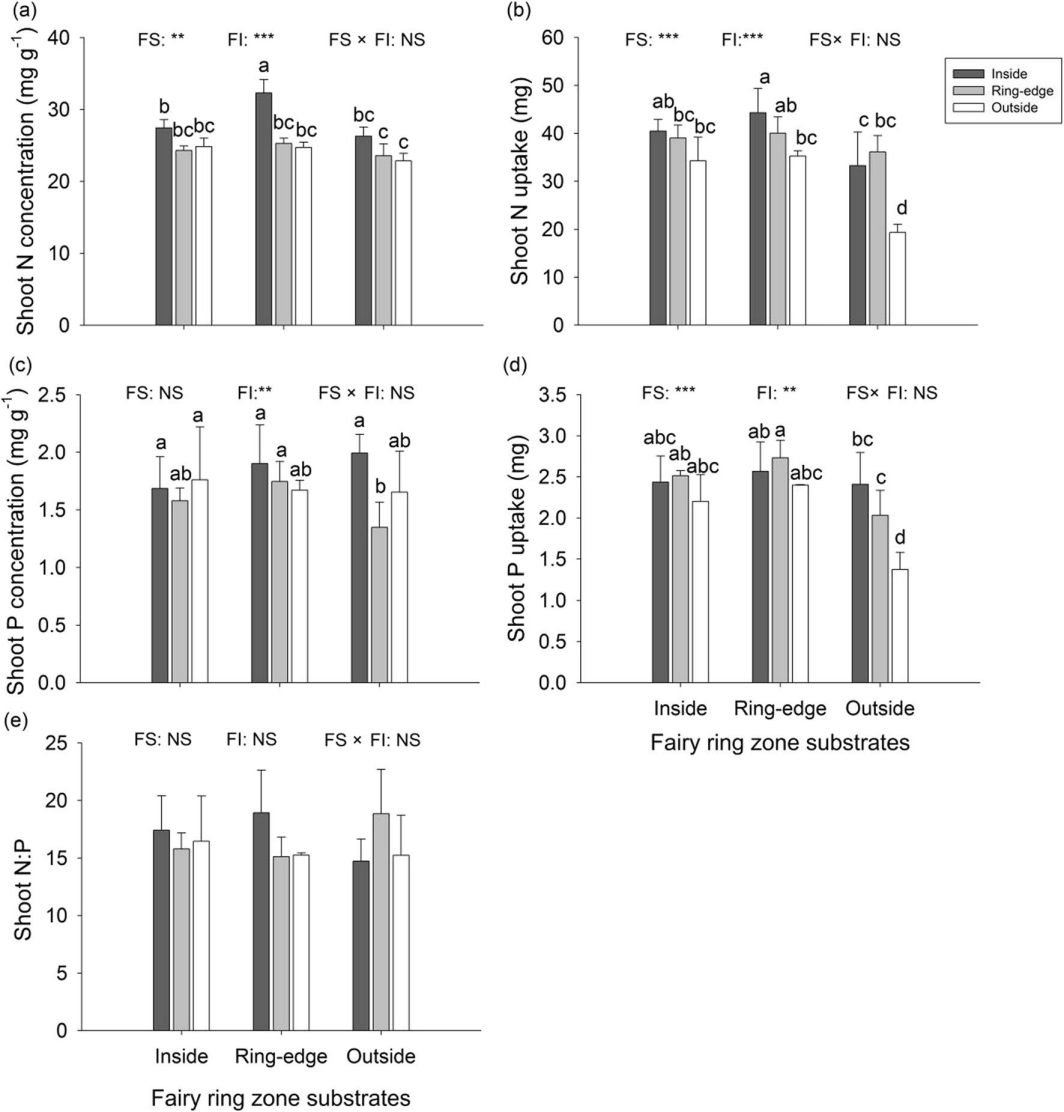
Shoot N concentration (**a**) and uptake (**b**), Shoot P concentration (**c**) and uptake (**d**), and N:P ratios (**e**) of *L. chinensis* grown in fairy ring zone substrates (collected from inside, ring-edge, and outside zones) inoculated with inside, ring-edge, or outside live soil (microorganisms) in greenhouse experiment. Error bar represent means + SE. Different letters indicate significant differences between treatments (*p* < 0.05), reported by Duncan’s multiple range test from one-way ANOVA

Fairy ring zone substrates and fairy ring zone inoculations both affected shoot N content and total shoot N uptake (Fig. 7a, b). Plants grown on inside and ring-edge substrates had higher shoot N concentration and shoot N uptake than on outside substrate (Fig. 7a, b). Inoculation with live inside soil increased *L. chinensis* shoot N concentration and shoot N uptake (Fig. 7a, b). Shoot P concentration was affected by microorganism origin. Living ring-edge soil decreased plant shoot P concentration, especially on outside soil substrate (Fig. 7c). Shoot P uptake was affected by both fairy ring zone substrates and fairy ring zone inoculations. Shoot P uptake was greater for plants growing on inside and ring-edge substrates. Soil inoculation from ring-edge or inside zones increased total shoot P uptake (Fig. 7d). Plant N:P ratio did not significantly differ among fairy ring zone substrates or fairy ring zone inoculations (Fig. 7e).

#### AM fungal colonization and root disease

**Fig. 8:**
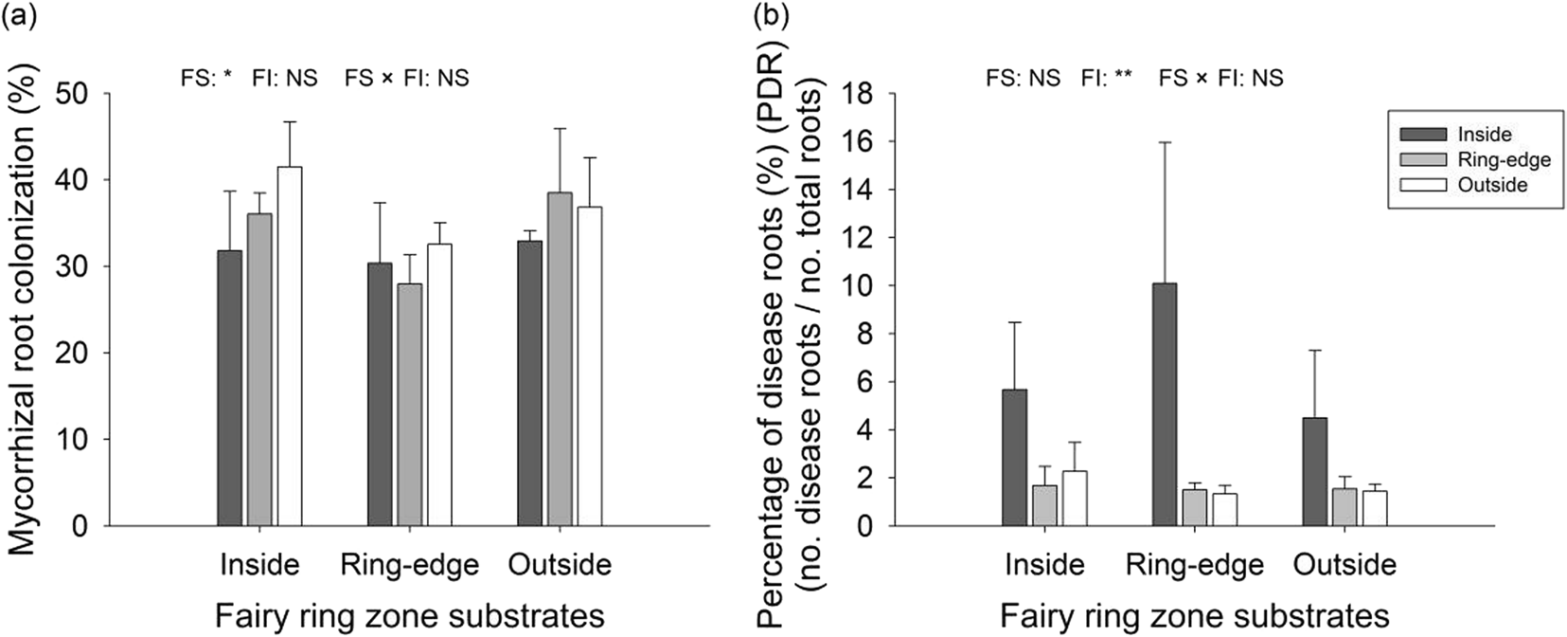
Arbuscular mycorrhizal (AM) fungal (**a**) and pathogen root colonization (**b**) of *L. chinensis* grown in fairy ring zone substrates (collected from inside, ring-edge, and outside zones) inoculated with inside, ring-edge, or outside live soil (microorganisms) in greenhouse experiment. Bars represent means + SE. Different letters indicate significant differences between treatments (*p* < 0.05), reported by Duncan’s multiple range test from one-way ANOVA

Arbuscular mycorrhizal fungal root colonization was significantly affected by fairy ring zone substrates (*p* < 0.05), with ring-edge substrates always characterized by lower mycorrhizal root colonization (Fig. 8a). Inoculant origin also significantly influenced root disease (*p* < 0.01). Inoculation with live soil collected from inside rings increased root disease in all three substrates, compared with microbial inoculation from soils collected at ring-edge or outside the ring (Fig. 8b).

## Discussion

Our results indicate soil microbial interactions drive fairy ring patterns in steppe grasslands dominated by *L. chinensis*, as *L. chinensis* growing on fairy ring-edges produced greater biomass compared to plants growing at the center of the ring or outside the ring (control). Our field study indicates this phenomenon is driven by microorganisms that mobilize soil P along the ring-edge. Further, our greenhouse experiment indicated microorganisms associated with the ring-edge stimulated the growth of *L. chinensis* directly through beneficial plant-soil–microbial interactions linked to live ring-edge inoculation, as well as indirect benefit through P mobilization. We speculate loss of plant production inside grassland fairy rings relates to loss of beneficial microorganisms and/or accumulation of pathogens, as inside fairy ring plants showed greater evidence of root disease in the field experiment. Furthermore, our greenhouse experiment showed live soil collected from inside fairy rings tended to cause more extensive root damage and lower shoot biomass when used as inoculum, compared to inoculum from ring-edges or outside the ring.

Considering abiotic soil conditions, optimal N:P ratios were considered the driving mechanism explaining fairy ring patterns in steppe grasslands [2]. However, we propose alterations in N:P ratios of fairy rings are strongly influenced by microbial ecology, rather than the driving mechanism of plant growth patterns. Plant growth and N:P ratio depend largely on soil fertility and homeostasis regulation [23]. In our field experiment, plant N:P ratio variation across fairy ring zones were not in line with soil plant-available N:P ratio in our field experiment. Soil N:P ratios were 4.96 (inside), 7.49 (ring), and 5.53 (outside), whereas the optimum N: P ratio for *L. chinensis* is close to 13.5 (Additional file 1: Fig. S1). However, we cannot attribute increased plant shoot biomass to the highest plant-available N:P ratio in ring-edge soil. Based on the results of our greenhouse experiment, although plant-available N:P ratio of outside soil increased after sterilization, shoot biomass of *L. chinensis* grown in outside soil was consistently lower compared to inside and ring-edge substrates. Therefore, we suggest that the increase of plant biomass in the ring-edge cannot be attributed only to N:P ratio.

Soil microorganisms play key roles in numerous nutrient processes including soil nutrient cycling and plant nutrient acquisition [24]. Microbes likely facilitate plant nutrient uptake at the ring-edge, leading to lush vegetation as fairy rings expand in grasslands. From our field results, soil microbial communities were highly variable across the three fairy ring zones. Zotti et al., reported a recovery of soil microbial communities, with expansion of the ring [10], however, our microbial communities remained stable following ring expansion. Soil microorganisms on ring-edge and inside zones were characterized by greater abundance of bacterial PLFA biomarkers compared to soils outside the rings, and these bacteria may promote P-solubilization. Bacteria solubilize and mineralize soil P and transform organic P into plant-available P [25]. A bacterial enzyme, alkaline phosphatase, is crucial for P-mineralization [26, 27]. Ring-edge and inside soil showed higher alkaline phosphatase activity compared to outside soil, and this may contribute to higher plant-available P concentrations in ring-edge and inside soil.

Plant–AM fungal interactions typically improve plant N and P uptake and enhance plant growth [28, 29]. We originally hypothesized the greater plant biomass production at ring-edge was derived from AM interactions. However, increased plant-available nutrients at the ring-edge and fairy ring center may have contributed to reduced AM fungal root colonization. Indeed, in the field, colonization of plants growing inside or along the ring-edge was reduced by > 300% compared to outside controls. We propose low nutrient availability of soils outside the rings promote AM fungal–plant interactions, and increased soil nutrient in ring-edge and inside fairy ring reduce the symbiotic activity. Alternatively, pathogenic fungi, as indicated by diseased root abundance, showed the opposite trend with greater presence of disease observed inside the rings. Reduction in mycorrhizal colonization may lead to increased abundance of diseased roots, as AM fungi may offer pathogenic protection of roots [8]. In our greenhouse experiment, ring-edge soil inoculation did not increase or decrease colonization of AM fungi but resulted in increased plant biomass, suggesting lush plants at fairy ring-edges are not driven by plant–AM interactions.

Although AM fungi did not play a substantial role in fairy ring patterns, inoculation with ring-edge soil did promote plant growth while decreasing shoot P concentration, especially on soil substrate collected from outside the rings. This indicates soil microorganisms may stimulate plant growth directly, not only through improved nutrient availability. For example, plant growth promoting rhizobacteria (PGPR) regulate plant growth and metabolism through phytohormones [30]. Soil bacteria, such as *Agrobacterium*, *Serratia*, and *Streptomyces* produce indole-3-acetic acid (IAA), a phytohormone that directly influences plant growth [31]. Choi et al. purified 2-azahypoxanthin (AHX) from fairy ring fungi (*Lepistasordida*) in turfgrass and found environmental stress and nitrogen absorption were improved by AHX[6].

Reduced plant production inside the fairy rings was likely influenced by several mechanisms, for example self-organization [32], phosphorus shortage [33] and decreases in exchangeable potassium (K) [34]. Self-organization theory predicts biomass–water feedbacks drive fairy ring patterns in water-limited systems. However, in our study, soil water content was not significantly different across fairy ring zone. We did not examine alterations in potassium among different fairy ring zones in our current study, but the role of exchangeable potassium in grassland fairy rings presents an interesting option for further study. Alternatively, models suggest vegetation patterns may be due to accumulation of negative plant-soil feedbacks in the center of fairy rings [13]. Dominant plants promote species-specific pathogens that inhibit conspecific growth [35]. Our results demonstrate inoculation with live soil collected from inside fairy rings reduced plant biomass. We attribute this response, at least in part, to pathogen accumulation based on increasing diseased roots, while Vincenot et al. hypothesized autotoxicity and Sheffer et al. (2007) suggested water depletion as the most important driving mechanisms [13, 36]. In our field investigation, *L. chinensis* growing inside fairy rings suffered up to 90% greater disease severity compared to plants growing at ring-edge or outside controls. Our greenhouse experiment provides further evidence, as inoculation with soil from inside the fairy ring resulted in abundant root diseases, regardless of fairy ring zone substrates.

## Conclusion

We provide empirical evidence that soil microorganism drive fairy ring vegetation patterns in semi-arid grasslands. First, as fairy rings expand across seasons, pathogens accumulate, with negative effects on the dominate species (*L. chinensis* in our study). Second, we found microorganisms in ring-edge soil may directly stimulate plant growth. Furthermore, these microorganisms were associated with greater alkaline phosphatase, promoting plant-available P-release in ring-edge soils. Additional biotic factors, such as AM fungal symbioses, may further explain dramatic above- and belowground differences observed across fairy ring zones, but did not appear to be a major influence in vegetation characteristics of *L. chinensis* across the three zones of fairy rings in our study. These insights expand our understanding of fairy rings, common but mysterious landscape features, highlighting essential links between vegetation dynamics and the microbial communities that shape grassland ecosystems. However, our findings should be viewed as a foundation for further research on fairy ring dynamics in grasslands. The fairy rings we assessed in the typical steppe of northern China suggest microbial interactions are linked with multiple plant community and soil food web outcomes and provides baseline data for several opportunities to build on by adding additional metrics, such as molecular identities of soil organisms, as well as to expand our results across additional grassland types. Only the dominant species (*L. chinensis*) in steppe grasslands was assessed in detail in our study, and the differences and driving factors of other species among fairy ring zones may help complete models of these complex and enigmatic ecological relationships.

## Supplementary information

**Additional file 1: Figure S1.** *Leymus chinensis* biomassproduction under different Stoichiometric ratios in greenhouse experiment.

## Data Availability

All data were summarized in the manuscript. Please contact the corresponding author regarding any additional queries related to datasets generated and analyzed for the current study, datasets are available from the corresponding author on request.

## Abbreviations

AM: Arbuscular mycorrhizal
AHX: 2-Azahypoxanthine
IAA: 3-Indole acetic acid
PMI: Plant–microbial interactions
TN: Total N
TP: Total P
AN: Plant-available nitrogen
AP: Plant-available P
APA: Alkaline phosphatase activity
FS: Fairy ring zone substrates
FI: Fairy ring zone inoculations
N: Nitrogen
P: Phosphorus
$${\text{NH}}_{4}^{+}$$: Ammonium
$${\text{NO}}_{3}^{-}$$: Nitrate
PLFA: Phospholipid fatty acid
NMDS: Non-metric multidimensional scaling
