# Supplementary material for: Assessing soil microbes that drive fairy ring patterns in temperate semiarid grasslands
Source: BMC Ecol Evol. 2022 Nov 5;22:130. doi: 10.1186/s12862-022-02082-x (PMC9636817; doi:10.1186/s12862-022-02082-x)
Supplement: Supplementary file 1 — Additional file 1: Figure S1. Leymus chinensis biomassproduction under different Stoichiometric ratios in greenhouse experiment. [file 12862_2022_2082_MOESM1_ESM.docx]

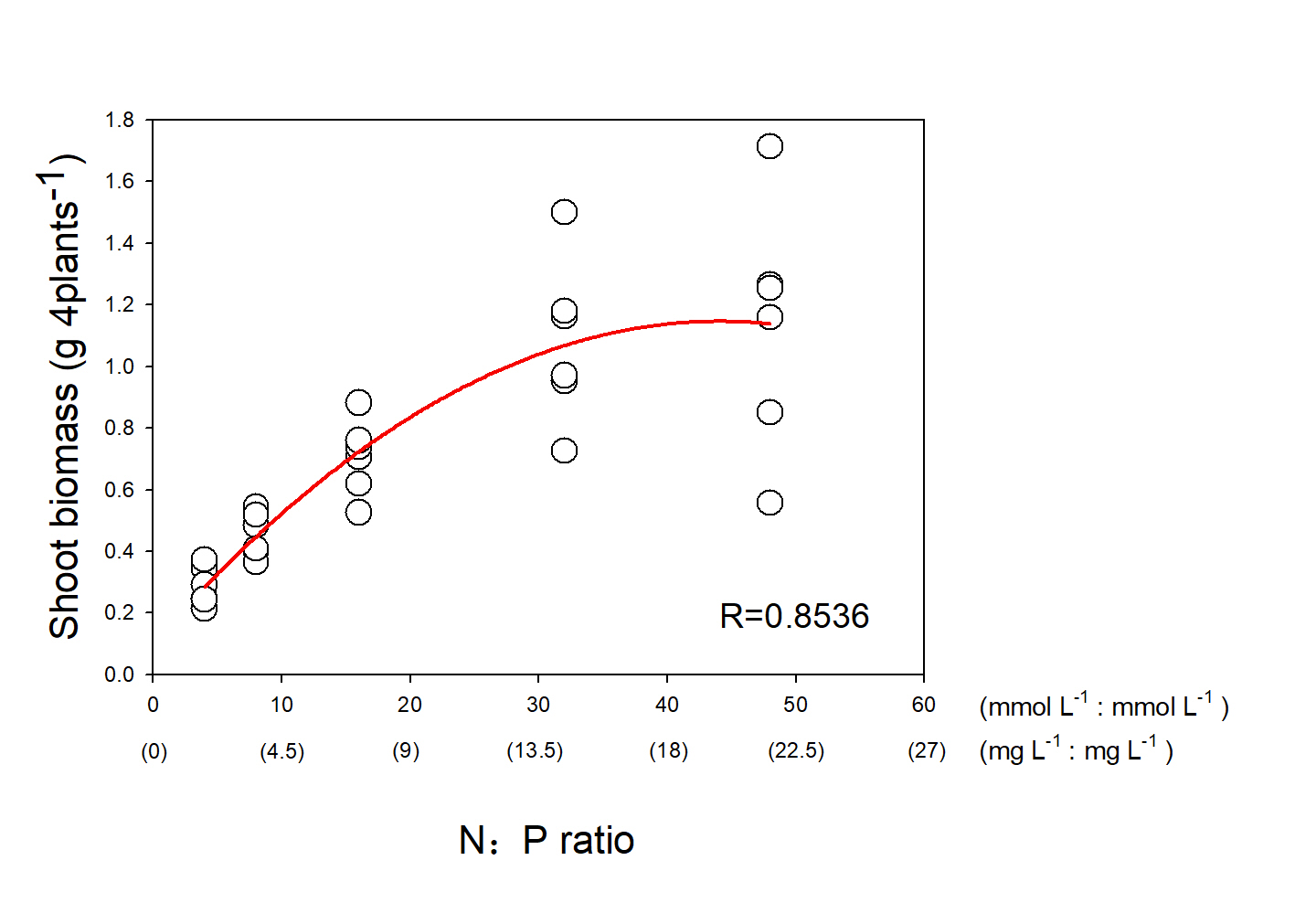


Appendix S1.

Figure S1 *Leymus chinensis* biomass production under different Stoichiometric ratios in greenhouse experiment.
